# Ten simple rules for creating a global network in computational biology

**DOI:** 10.1371/journal.pcbi.1010528

**Published:** 2022-10-27

**Authors:** Jenea Imani Adams, Taylor Ferebee, Melyssa Minto, Kayla K. Pennerman, Nyasha Chambwe

**Affiliations:** 1 Genomics and Computational Biology Graduate Group, University of Pennsylvania, Philadelphia, Pennsylvania, United States of America; 2 Department of Computational Biology, Cornell University, Ithaca, New York, United States of America; 3 Computational Biology and Bioinformatics, Duke University, Durham, North Carolina, United States of America; 4 Joint Institute for Food Safety and Applied Nutrition, University of Maryland, College Park, Maryland, United States of America; 5 Institute of Molecular Medicine, The Feinstein Institutes for Medical Research, Northwell Health, Manhasset, New York, United States of America

## Introduction

The emergence of large-scale biological data is transforming the way that we advance impactful science to change the world around us for good. Unfortunately, the benefits of large-scale data are not shared equally by all communities. People of African descent are not merely “historically marginalized communities.” They are “intentionally segregated, underserved, and disenfranchised communities” [1]. Black researchers are more likely to engage in research that directly impacts their communities [2], health data, food systems data [3]. The biomedical workforce remains disproportionately centered around Western white populations. Black researchers are also among the least-funded demographic group across several professional levels [2,4], especially within computationally oriented biomedical research [5]. Consequently, research that affects Black communities is implicitly devalued within the academic ecosystem. Topics in biological research, including genomics and precision health, need urgent attention from data science practitioners who can continue to aid the translation of this research to communities in need. To address this need, computational biology must cultivate and support minoritized researchers who confront anti-Black racism and sexism in this field.

The systemic and structural barriers that Black women face in science, technology, engineering, and mathematics (STEM) dissuade many from pursuing the required studies for their academic career. These barriers include systemic bias in how academia measures success, including conferral of advanced degrees, publishing, funding, and prestigious awards. Only 33% to 45% of people who earned US doctorates in computational biology or bioinformatics in 2020 are women [6]. In 2020, only 7 non-Hispanic Black people graduated with a doctoral degree in computational biology or bioinformatics in the United States (US) compared to non-Hispanic white counterparts who consistently receive upwards of 100 to 200 US doctorates per year [6]. Women are underrepresented in computational biology publications compared to biology more broadly, independent of the journal’s impact factor [7,8]. The International Society for Computational Biology (ISCB), computational biology’s leading professional society, is twice as likely to confer honors on US-affiliated scientists than expected based on authorship demographics in the field [7].

Black women, members of 2 or more marginalized groups, are noticeably absent from celebrated spaces and groups in computational biology. For example, ISCB lacks gender and ethnic membership diversity [9], which impacts opportunities for awards, scientific presentations, and networking. To combat systemic exclusion in this field, we champion the role of social support networks for racially minoritized women. Networks provide a source of support and camaraderie that can contribute to long-term persistence in STEM [10]. These networks can impact their members at all career stages by providing resources to advance their training and education, as well as by expanding individual networks for support in job searches, finding collaborators, and identifying mentors. Networks can elevate and amplify the achievements of minoritized individuals to increase their visibility and increase opportunities available to them.

**Fig 1:**
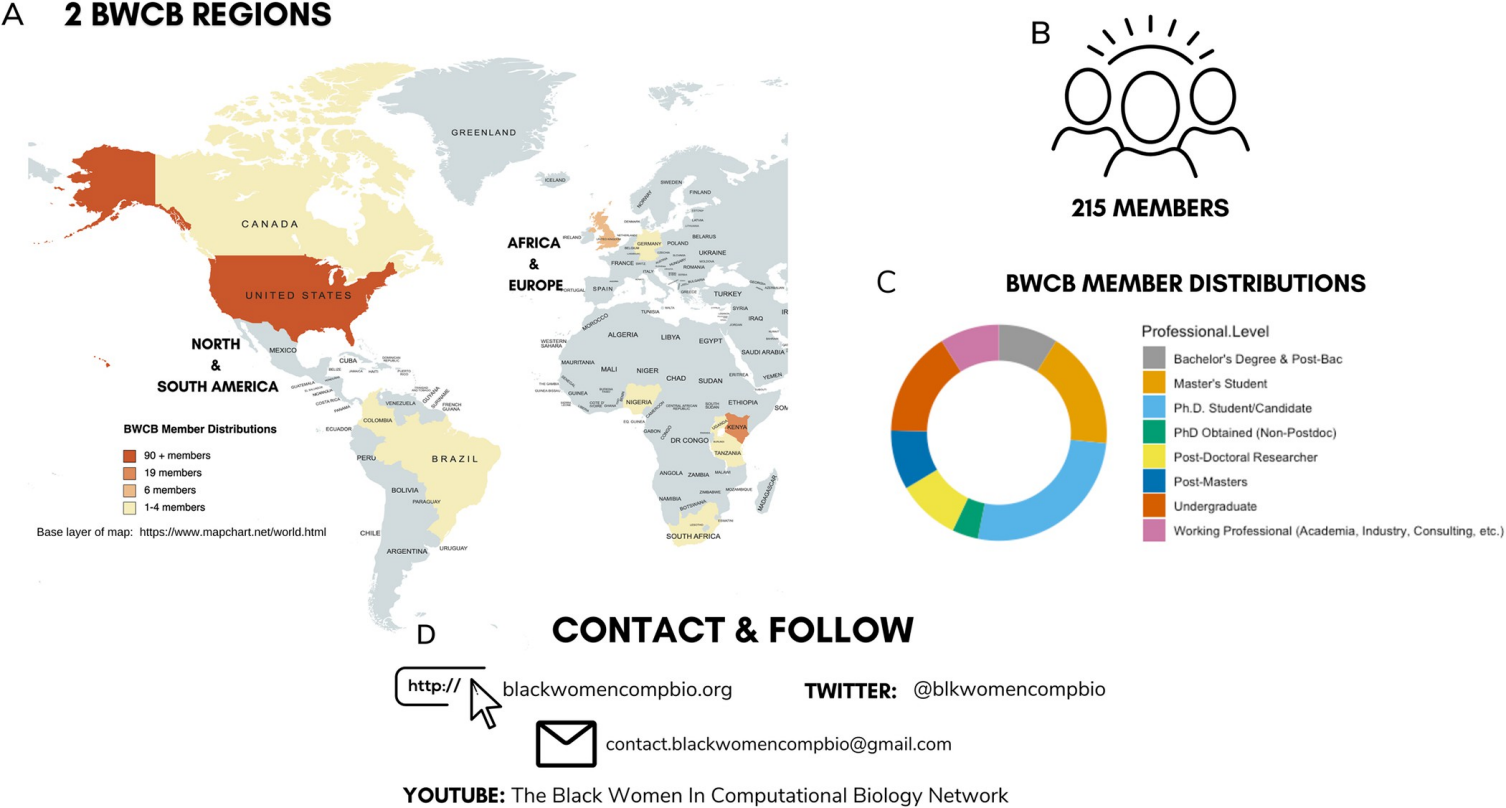
Statistics and contact information for the Black Women in Computational Biology Network (BWCB). (**A**) BWCB currently has 2 regions, encompassing the continents of (1) North and South America; and (2) Africa and Europe. (**B**) There are currently 215 members. (**C**) Demographic summary of BWCB members’ professional levels. (**D**) Contact information. Members stay connected through the website (blackwomencompbio.org), Twitter (@blkwomencompbio), email (contact.blackwomencompbio@gmail.com), and YouTube (The Black Women in Computational Biology Network).

**Fig 2:**
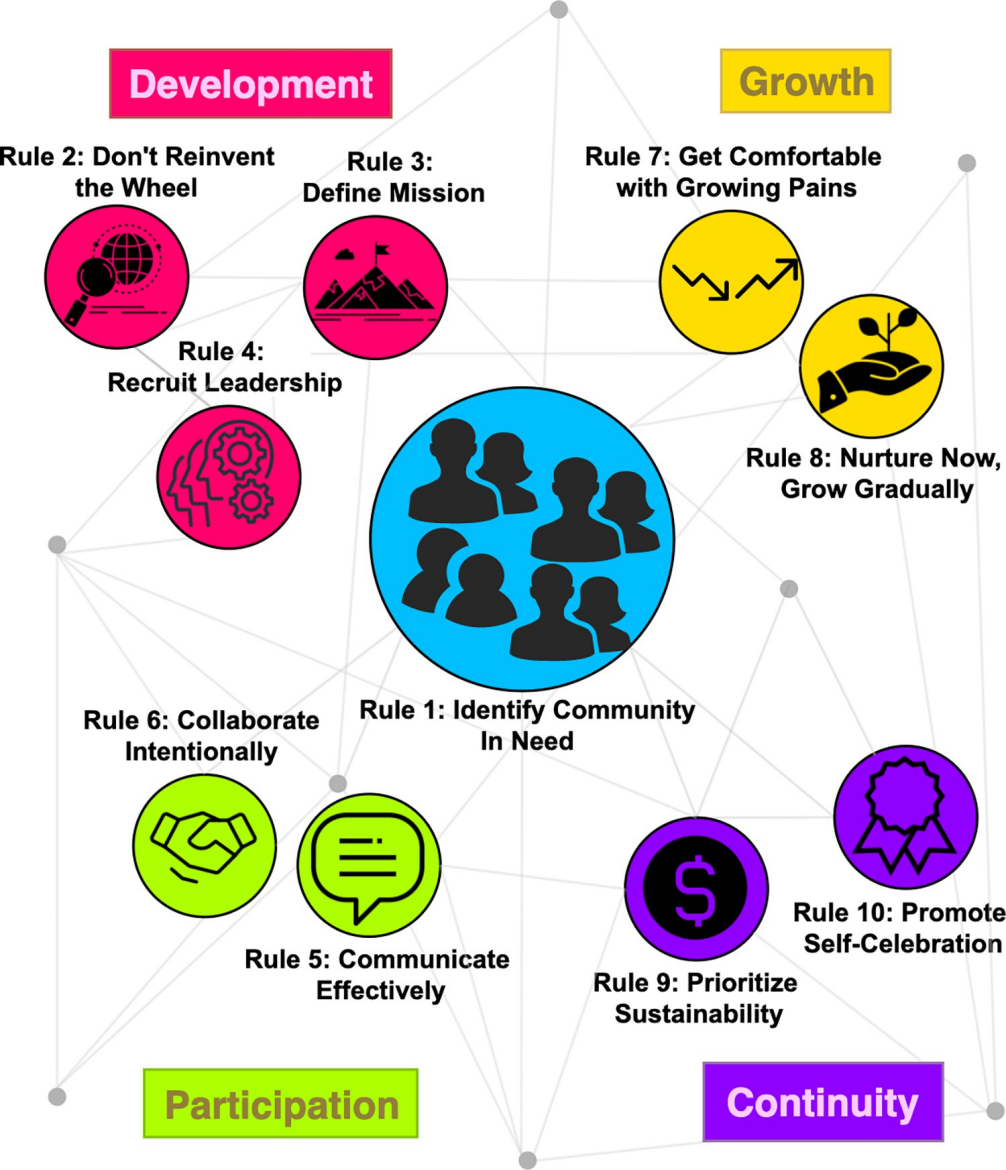
Network diagram summarizing the Ten Simple Rules for Creating a Global Network in Computational Biology. **Blue** center node focuses on identifying an isolated community (Rule 1). **Pink nodes** describe the building blocks of the global network development process: the importance of looking to other organizations for inspiration (Rule 2), defining a mission (Rule 3), and recruiting leadership (Rule 4). **Green** nodes relate to the group’s external and internal participation, including the need for effective communication (Rule 5) and intentional collaboration (Rule 6). **Yellow** nodes, associated with organizational growth, outline getting comfortable with inevitable roadblocks (Rule 7) and fostering communities with the goal of gradual growth (Rule 8). **Purple** nodes describe the continuity-based practices that ensure group sustainability (Rule 9) and promote the self-celebration of members (Rule 10). The nodes are interconnected, highlighting the iterative nature of the rules as the group grows and changes over time.

To that end, we created the Black Women in Computational Biology (BWCB) Network in 2020 to increase the visibility of Black women in the field of computational biology and to preserve and advocate for their scientific identities (**Fig 1**). Since its formation, BWCB has grown from a small collective to an international pillar of the computational biology community, with over 200 members representing 4 continents (**Fig 1**). In this article, we outline a framework for bringing together communities to combat systemic exclusion in computational biology (**Fig 2**). We strive to inspire leaders who embody intersectional identities to create avenues for professional and interpersonal growth and the development of a more diverse computational biology field. We hope that this, in turn, will create a community-centric approach for improving the use of computational approaches to advancing biological research.

### Rule 1: Identify a community in need of connection

Identify a demographic group with unmet support in the field. It is crucial to confirm that there is a need for and sufficient interest to sustain your intended community. The basis for creating a professional organization for minoritized scientists is finding an area in which something is lacking or needs improvement, a process not unlike forming a new research question. Evaluate the identities of people within the field and determine if any are underrepresented or in need of streamlined networking and connection-building efforts.

In our case, Jenea Adams, a current PhD candidate in computational biology, realized that Black women were not adequately represented in her field. She was often the only Black woman on the PhD interview trail or in her doctoral classes, and she could only name about 3 other Black women who she knew in the field. In contrast, her non-Black peers had access to myriad people who understood how their cultural, social, and demographic identities intersected with their professional lives. Black women do not share this experience, which can have tangible consequences such as making it more difficult to find mentors or a support network to help navigate academic and professional environments. The cycle of lack of representation and systemic racism fueling poor mental health, which can present as imposter syndrome, stereotype threat, and heightened levels of anxiety, stress, and depression [4,11–13], starts early on. The adverse risks associated with poor educational, social, and cultural ties are significantly lowered in Black teenagers who have access to relatable Black female mentors [14]. These mentoring relationships also have psychological gains for mentors [14–15]. Many of these ideologies of culturally responsive mentorship and a sense of belonging are rooted in foundational Black feminist scholarship and tie into sociopolitical foundations of social capital [16]. For these reasons, BWCB emerged through a shared desire for connection among members. BWCB’s goal is to create a safe space for Black women to make the integral connections necessary to achieve their professional goals. We work to increase the visibility of those who identify as Black female computational biologists and to build community across institutions and borders. This network brings tremendous benefits to a population of computational researchers from historically excluded backgrounds. With adequate support, these Black women will be able to contribute their expertise and interests to help their communities more broadly.

### Rule 2: Don’t reinvent the wheel

In developing your community, identify and research comparable groups (both established and newer) with missions similar to your own. This research can be used to formulate the key determinants for success in building your network. Develop a framework that condenses the best ideas from comparable groups while addressing your group’s unique mission, target audience, and desired impact to fill a gap in need. In forming BWCB, we looked at several established groups with exemplary qualities (Table 1).

**Table 1:** Key professional networks and organizations considered in developing the BWCB Network.

Quality	Organization	Quote
Mission	Disabled in Higher Ed, Black and Disabled Discord Community (https://disabledinhighered.weebly.com/)	“We seek to amplify a perspective that is often silent and unseen.”
Audience	National Society of Black Engineers (https://www.nsbe.org/)	“supports and promotes the aspirations of minority collegiate and pre-collegiate students and technical professionals in engineering and technology.”
Impact	Society for Advancement of Chicanos/Hispanics and Native Americans in Science (https://www.sacnas.org/)	“317 Leaders Trained Since 2009… 5,861 Research Presentations Since 2015… 28,000+ Community of Supporters”
Novelty	STEMNoire (https://www.stemnoire.org/)	“. . .first-of-its-kind research conference and holistic wellness retreat for women of the African diaspora in the fields of [STEM]”

### Rule 3: Define the mission

An organization’s mission statement encompasses the central set of values that guides its emphases, resources, goals, and time. The mission is the glue that holds a group together. For communities with members from different research areas, the mission statement outlines commonalities and shared goals. When crafting the mission, think critically about what makes your group unique, and what your areas of focus are (e.g., action and advocacy, networking, science communication). Thinking about your mission proactively and critically will help you to refine your voice and identify areas where your organization can be most impactful for the community you serve. As the group evolves, your mission statement may need to be refined to reflect its changing priorities and goals more accurately.

At present, BWCB’s areas of collective action are the following:

- **Resource amplification**: to create and share access by creating opportunities, pathways, and resources among network members
- **Science communication**: to broaden participation and actively support the success of Black women by increasing awareness of the computational biology community
- **Intergenerational collaboration**: to contribute favorably to vibrant tiered mentoring opportunities and diverse interactions by engaging members from a wide range of stages in their careers
- **Critical scientific engagement**: to create a safe space to engage and learn from one another as scientists by hosting journal clubs, research talks, and seminars for Black women to develop as scientists
- **Global citizenship perspective**: to acknowledge, express, and welcome the richness of the broad diaspora of Black women scientists from different cultural, social, educational, and economic backgrounds throughout the world.

### Rule 4: Recruit a committed leadership team

A successful organization has leaders who ensure its growth, development, and sustainability. The leadership should comprise reliable individuals who are experienced in the relevant field, committed to the organization’s goals, as well as representative of and respected by the membership. In addition to internal leadership, consider an external advisory board composed of individuals that have experience in supporting similar communities to help guide the overall direction of the group and point to key trends in the field. To ensure team success, effective communication and cooperation skills are must-haves [17]. There are 4 critical decisions to make upfront about the network’s leadership team: structure, selection, support, and succession. However, don’t fear change!

#### Structure

Lay the foundation for how the network will run. In BWCB, the leadership team oversees the organization’s main goals in permanent committees that are dedicated to the following:

- **Membership:** to onboard new members and connect them to relevant BWCB spaces
- **Community engagement:** to form and maintain partnerships with other organizations that expand the network and resources available for BWCB members
- **Seminars, workshops, and programming:** to plan and host monthly seminar series and other events
- **Regional representation:** to advise and oversee the allocation of resources and event planning between the 2 broad regions represented in our membership

#### Selection

How leaders are chosen will affect leadership composition. There are pros and cons to democratic elections versus appointments versus some hybrid of the two. Your group may benefit from utilizing a preselection process that focuses on finding individuals with appropriate skill sets, valuable ideas, and compatible personalities before an election or appointment. Since computational biology encompasses a broad range of subfields, it was important for BWCB to recruit a team that represents various domains of computational biology to ensure unbiased programming. Make sure your selection criteria take unique aspects of your discipline and target audience into consideration.

#### Support

Long-term maintenance of the leadership helps to sustain the organization. This involves the communication of expectations, regular meetings, and progressive work toward consensus and trust in each other. No true community is a one-person show, and neither is true leadership.

#### Succession

Good leadership teams have turnover. You should make a plan for how to maintain continuity and efficiency as the team shifts. Strategies for succession include having overlapping terms or transition periods where former officers train incoming officers, writing or updating protocols for all tasks, and keeping in contact with former officers. Record everything from the ideation and planning to the execution of functions and events. When new leaders take over, they should have a successful template from which to build.

### Rule 5: Communicate effectively

Communication is critical for building the group’s reputation and identity, as well as for fostering relationships. As computational biologists, we are often familiar with varying subdiscipline-specific jargon in our work. We are also less likely to be confined to physical laboratories to perform our jobs productively. Given this fluidity in communication, your network will need to convene and disseminate information in effective, flexible, and sustainable ways. Organizational communication has 4 essential functions: (1) to support internal and external operations; (2) to build reputation and identity; (3) to foster trusted relationships with stakeholders; and (4) to listen for environmental changes [18]. Group leadership must communicate tasks internally, share information on meetings, log agendas, and keep good notes.

Platforms that support internal chats, external communication, or cloud-based storage provide great tools to build and maintain connections while enabling archiving of essential information. An organization’s website is a key component of external communications. Many services provide free to low-cost website templates for delivering public and membership-specific information. Highly interactive social media platforms in widespread use in academic spaces can be instrumental in forming an audience that identifies with your mission and vision. Newsletters sent at consistent intervals using email automation services can promote events, showcase sponsors, and introduce readers to organization members.

Consider accessibility in all communications. While many popular software platforms in computational biology are offered in English, that may not be everyone’s first language. Consider using platforms that offer automatic translations of your material and accessible infrastructure (i.e., alternative text, captions on video meetings, color blind–friendly palettes) to ensure that your communications can be enjoyed by all. Be mindful of the group’s communication values as you set development goals and evaluate how effectively your communication strategies help with growth.

### Rule 6: Collaborate intentionally

Recognize that key resources and knowledge needed to serve your community may come from outside the group. Think critically about who to engage and partner with, and think about how external contributors support the mission, vision, and goals of your organization. This support can take the form of allyship, the long-term commitment that someone with power makes to the demarginalization of a disempowered group through active promotion [19]. Allyship is a great way to provide inclusive opportunities for engagement with a broad range of people. We found that it is vital to approach allyship as a role that is centrally defined by the group being served, not by those interested in being an ally.

BWCB has established a supporter network that includes individuals who contribute resources or their expertise to the group. Larger communities, such as PLOS Computational Biology, ISCB, American Medical Informatics Association, Women in Data Science, and H3ABioNet, have the platforms to sponsor BWCB members in different ways. We ask new supporters to specify how they would like to get involved in our community to provide an accessible way for our members to find resources and connections outside the network for their continued career development. We are intentional about reaching out to our supporters to help amplify a cause within the BWCB network, for example, looking for new research opportunities for members, donating to maintain our website, attending events, and engaging in our seminar series.

Partnerships require balance. Be protective of your mission and your members in a way that centers your membership’s voice in crafting your narrative. Performative allyship is a form of personal gain at the expense of the central community being served, rather than true devotion to a cause or the people who should be amplified [20]. Be wary of performative allyship—especially if your network serves persons who are historically excluded from broader participation in computational biology.

### Rule 7: Get comfortable with growing pains

Change is inevitable. As your organization grows and science advances, ideas and values may shift as different tools, platforms, and interests change in popularity. While an individual leader may have a unique vision for the group, the group’s success is ultimately driven by the people served. Therefore, it is crucial to remain aware of these shifts by regularly querying the group’s members on how they would like to be supported.

To keep up with rapidly evolving developments in the computational biology field, BWCB supports members’ professional development. For example, through our recent journal club discussions, we discovered that members had an interest in implementing the tools we were discussing. We collectively came up with the idea of participating in team-based bioinformatics research challenges. These will allow members, independent of their location, to collaborate and showcase skills based on their interests, preparing them for work in their own disciplines. As interests in the group change, try leading new initiatives that fill the gaps between what the group offers versus what the group needs.

It is important that leadership adopt a growth mindset and view setbacks as learning moments [21,22]. Planning and running network operations take substantial effort. It is easy to make mistakes as the logistics behind successfully curating a thriving, sustainable group are nontrivial. Recognize that we all make mistakes and be willing to adapt as your group grows. Finally, be open to criticism and be prepared to incorporate suggestions for improving the group. Ultimately, a shared commitment to your group’s mission will get you through any challenge.

### Rule 8: Nurture now, grow gradually

As your community evolves, nurture the organization and its structure. For us, such nurturing includes providing a space for new members to be welcomed and actively integrated into our community, reaching out to future members, and raising community awareness. A “space” is a monitored, protected, but intentional virtual or physical entity for communication within a subgroup. Do not neglect any of these aspects, as they help everyone to feel seen, heard, and valued. In BWCB, members are encouraged to contribute at each stage of the organization’s evolution. We also host networking events that are easily accessible to new members and are an excellent way for current members to reconnect and meet new colleagues and friends. Balancing the goals and values of your community and leadership team is at the heart of your organization.

### Rule 9: Prioritize sustainability

As networks are intended to provide long-lasting professional connections, you want your community to last for more than a season. Sustainability must be the foundation of your organization. To prioritize the sustainability of your organization, consider funding, business incorporation, and strategic growth.

Maintaining an effective platform for mass community building and professional development is not free. There are significant additive costs to website hosting and domain names, newsletter/subscriber management, online file storage, social media marketing, and honorariums and gifts for seminar speakers. No individual member should be responsible for these costs. Instead, consider creating a business account with an online payment vendor that has donation portals that enable safe, secure, and automated fundraising and logging of all the organization’s financial activities across many of the world’s currencies. Obtain an associated account-specific debit card, so that the organization can pay vendors that do not allow virtual payments, while simultaneously tracking expenses for accounting purposes. Beyond passive fundraising, you might consider monetizing your job board or certain public events, although BWCB currently offers these spaces and services at no cost.

Consider incorporating your organization as a nonprofit or similar entity to formalize fundraising activities. Incorporation makes your network eligible for a wider array of grants, provides a framework for official sponsorship, and allows your organization to be acknowledged as a legitimate, federally recognized network. For example, starting a nonprofit in the US requires the investment of a consistent leadership team or advisory board willing to document their involvement with the organization. These requirements are in place because the essence of a nonprofit is that no sole individual “owns” the organization. Incorporation costs are high, upwards of US$1,000, depending on the registered geographic location of the organization. Incorporation requires special consideration but could be a great fit for leaders looking to grow on a larger scale.

It is important to effect these sustainability measures early on to ensure the long-term success of the network. Create a protocol for expenses and budgeting. Consistently using this protocol will ensure that documentation is never lost as the organization grows and leadership changes. Identify potential board members for incorporation or long-term advising in advance. Board members might include individuals who demonstrate high organizational commitment, provide extra service, and express interest in long-term leadership engagement in the network. Early consideration will give you sufficient time to build relationships of trust with these individuals as you move forward with making critical decisions for your fledgling organization.

### Rule 10: Promote self-celebration

Celebrate the group’s achievements. This rule satisfies one reason for why BWCB was established: to provide members with more chances for wider recognition of their work. Self-celebration can include encouraging enrollments in open competitions and nominating each other for awards. However, a group can create its own opportunities by actions such as honoring seminar presenters for great talks or hosting hackathons.

BWCB members regularly celebrate each other with member highlights on our social media channels, through our internal message board, and via our monthly member newsletter. We provide opportunities to share each other’s research and scientific interests in biregional journal clubs and network-wide summits. Additionally, we invite notable Black computational biologists to our #BlackInCompBio Seminar Series to showcase their science to our global community and encourage scientific engagement (see Rule 3). We are here to extend the spotlight to include more Black women in our field.

## Conclusions

Computational biology is a rich, rapidly growing field that is changing the pace and rigor at which impactful science is advanced. Many applications of this interdisciplinary field benefit from diverse perspectives and the amplification of voices historically excluded from interdisciplinary science. One way to broaden participation and retention of minoritized scientists in this field is through the creation of professional networks. This is evidenced by the growth and success of the BWCB Network, which connects our members to mentoring, professional development, training, and community-building resources that are vital to their growth and development as Black woman scientists.

While the creation of BWCB has impacted many of Black women in the field, there is a large need for the continued formation of similar organizations to support other communities that are underrepresented in the field. A recent study showed that, even with existing support systems, the gender gap will persist in computational biology and its related subfields for the next 25+ years [23]. This can be attributed to the slow pace of academic institutions’ efforts to recruit and retain women within these fields. We note that rapid change in academia can occur as seen in response to the COVID-19 pandemic. In short order universities have managed to largely pivot to online learning, waive tuition in some cases, and, yet, have been unable to make impactful changes toward racial or gender equity [24]. More organizations that serve historically marginalized groups are needed to propel the closing of these equity gaps.

Creating a successful network requires strong communication and organization, spaces to facilitate meaningful connections, and most importantly, a clear mission to benefit a target population. Weaving sustainability into the group’s genesis and planning will help inform the group structure that would best serve those goals. While there will be trials and errors in the quest to fulfill the mission, you will eventually learn what works best for your group. What you create has the potential to be a safe refuge and a place for connection and intellectual encouragement. Keep the end goal in mind as you persevere through startup and growing pains as the beneficial impacts of this group will far outweigh and outlast the temporary struggles. Ground-breaking science is not done in a vacuum. Building communities that help scientists realize and actualize their best selves creates a brighter future for all. We share our experience here in hopes that computational biology continues to include and uplift minoritized scientists within the field. We encourage all STEM groups to use similar frameworks.

## References

[1] Watson T. “Historically marginalized communities…” Nah. “Intentionally segregated, underserved and disenfranchised Black and Latinx communities…” #WritingDay. https://twitter.com/terrinwatson/status/1504075321859092480. Posted 2022 Mar 16.

[2] Taffe MA, Gilpin NW. Racial inequity in grant funding from the US National Institutes of Health. Elife. 2021 Jan;18:10. doi: 10.7554/eLife.65697 33459595 PMC7840175

[3] Carter AL, Alexander A. Soul Food: [Re]framing the African-American Farming Crisis Using the Culture-Centered Approach. Front Commun. 2020 Feb 18;5.

[4] Davies SW, Putnam HM, Ainsworth T, Baum JK, Bove CB, Crosby SC, et al. Promoting inclusive metrics of success and impact to dismantle a discriminatory reward system in science. PLoS Biol. 2021 Jun 15;19(6):e3001282. doi: 10.1371/journal.pbio.3001282 34129646 PMC8205123

[5] Stevens KR, Masters KS, Imoukhuede PI, Haynes KA, Setton LA, Cosgriff-Hernandez E, et al. Fund Black scientists. Cell. 2021 Feb;184(3):561–5. doi: 10.1016/j.cell.2021.01.011 33503447

[6] (NCES) NC for S and ES. Doctorate Recipients from U.S. Universities: 2020 | NSF—National Science Foundation [Internet]. ncses.nsf.gov. 2021. Available from: https://ncses.nsf.gov/pubs/nsf22300

[7] Le TT, Himmelstein DS, Hippen AA, Gazzara MR, Greene CS. Analysis of scientific society honors reveals disparities. Cell Systems. 2021 Sep;12(9):900–906.e5. doi: 10.1016/j.cels.2021.07.007 34555325

[8] Bonham KS, Stefan MI. Women are underrepresented in computational biology: An analysis of the scholarly literature in biology, computer science and computational biology. Bergstrom CT, editor. PLoS Comput Biol. 2017 Oct 12;13(10):e1005134. doi: 10.1371/journal.pcbi.1005134 29023441 PMC5638210

[9] Equity, Diversity and Inclusion (EDI) Committee of the International Society for Computational Biology (ISCB). September 09, 2021: ISCB Releases Inaugural Equity, Diversity, and Inclusion Report [Internet]. [cited 2022 Sep 12]. Available from: https://www.iscb.org/iscb-news-items/4738-2021-sept09-iscb-releases-inaugural-equity-diversity-inclusion-report

[10] Turetsky KM, Purdie-Greenaway V, Cook JE, Curley JP, Cohen GL. A psychological intervention strengthens students’ peer social networks and promotes persistence in STEM. Sci Adv. 2020 Nov 6;6(45):eaba9221. doi: 10.1126/sciadv.aba9221 33158856 PMC7673703

[11] Dzirasa K. Revising the a Priori Hypothesis: Systemic Racism Has Penetrated Scientific Funding. Cell. 2020 Oct;183(3):576–9. doi: 10.1016/j.cell.2020.09.026 33125883

[12] Odekunle EA. Dismantling systemic racism in science. Sills J, editor. Science. 2020 Aug 13;369(6505):780.3–781. doi: 10.1126/science.abd7531 32792390

[13] Liu S-NC, Brown SEV, Sabat IE. Patching the “leaky pipeline”: Interventions for women of color faculty in STEM academia. Arch Sci Psychol. 2019 Nov 25;7(1):32–9.

[14] Lindsay-Dennis L, Cummings L, McClendon SC. Mentors’ Reflections on Developing a Culturally Responsive Mentoring Initiative for Urban African American Girls. Black Women, Gender + Families. 2011;5(2):66.

[15] Brown RN. Black girlhood celebration: toward a hip-hop feminist pedagogy. New York: Peter Lang; 2009.

[16] Solomon A, Moon D, Roberts AL, Gilbert JE. Not Just Black and Not Just a Woman: Black Women Belonging in Computing. 2018 Research on Equity and Sustained Participation in Engineering, Computing, and Technology (RESPECT). 2018 Feb:1–5. doi: 10.1109/RESPECT.2018.8491700

[17] Woolley AW, Aggarwal I, Malone TW. Collective Intelligence and Group Performance. Curr Dir Psychol Sci. 2015 Dec;24(6):420–4.

[18] Zerfass A, Viertmann C. The communication value circle. Communication Director [Internet]. 2016 May 30 [cited 2022 Sep 12]. Available from: https://www.communication-director.com/issues/unwritten-contract-social-licence-operate/communication-value-circle/#.Yx6DNexuf0o

[19] Bilal M, Balzora S, Pochapin MB, Oxentenko AS. The Need for Allyship in Achieving Gender Equity in Gastroenterology. Am J Gastroenterol. 2021 Dec 1;116(12):2321–2323. doi: 10.14309/ajg.0000000000001508 34665160

[20] Erskine SE, Bilimoria D. White Allyship of Afro-Diasporic Women in the Workplace: A Transformative Strategy for Organizational Change. J Leadersh Org Stud. 2019 May 15;26(3):319–38.

[21] Boomer J, direnpramodacumar. 6 Steps for Adopting a Growth Mindset [Internet]. CPA Practice Advisor. 2020 [cited 2022 Sep 12]. Available from: https://www.cpapracticeadvisor.com/2020/02/06/6-steps-for-adopting-a-growth-mindset/36250/

[22] Dweck CS. Mindset: The new psychology of success. New York: Ballantine Books; 2006.

[23] Holman L, Stuart-Fox D, Hauser CE. The gender gap in science: How long until women are equally represented? Sugimoto C, editor. PLoS Biol. 2018 Apr 19;16(4):e2004956. Available from: https://www.ncbi.nlm.nih.gov/pmc/articles/PMC5908072/29672508 10.1371/journal.pbio.2004956 PMC5908072

[24] Montgomery BL. Make equity essential to expedite change in academia. Nat Microbiol. 2020 Dec 21;6(1):7–8. 10.1038/s41564-020-00845-0 33349679

